# Advances in the extracellular vesicles treatment of alopecia

**DOI:** 10.1080/07853890.2025.2543517

**Published:** 2025-08-06

**Authors:** Xiaoxin Wu, Songjia Tang

**Affiliations:** a State Key Laboratory for Diagnosis and Treatment of Infectious Diseases, National Clinical Research Centre for Infectious Diseases, The First Affiliated Hospital, Zhejiang University School of Medicine, Hangzhou, Zhejiang, China; b Plastic and Aesthetic Surgery Department, Affiliated Hangzhou First People’s Hospital, School of Medicine, Westlake University, Hangzhou, Zhejiang, China

**Keywords:** Alopecia, extracellular vesicles, challenges, advances

## Abstract

**Background:**

Alopecia is a prevalent condition that significantly impacts appearance, mental health, and overall quality of life. Current clinical treatments for alopecia include medication, hair transplantation, low-level light therapy, and stem cell therapy, among others. However, the effectiveness of these treatments remains far from ideal. Extracellular vesicles (EVs) have emerged as a promising therapeutic strategy, yet challenges, such as mass production and clinical application persist. This review aims to explore recent advancements in the use of EVs for the treatment of alopecia.

**Methods:**

A comprehensive literature search was conducted in the PubMed database using a range of relevant keywords: extracellular vesicles, alopecia, hair loss, preparation, exosomes, microvesicles, apoptotic bodies, nanoscale particles, preclinical studies, clinical trial, clinical transformation, regenerative medicine, tissue engineering, stem cell, bioactive molecules, and extraction methods. The papers mostly focus on the biological characteristics and preparation of EVs, the basic and clinical research of EVs in the treatment of hair loss, and the challenges of clinical transformation of EVs were retracted.

**Results:**

EVs are nanoscale, spherical particles enclosed by a lipid bilayer. They exert their effects by activating signaling pathways through ligand binding or by entering cells *via* membrane fusion or endocytosis. Compared to traditional medications or stem cell therapies, EVs offer several advantages, including high biological safety, sustained efficacy, low risk of immune rejection, and ease of storage and transportation. These properties make them a highly promising therapeutic option.

**Conclusion:**

Significant progress has been made in understanding the preparation, mechanisms, and early clinical applications of EVs. However, further research is needed to optimize key factors, such as dosing, cell sources, and administration frequency to develop more effective and safer therapies. With continued innovation in manufacturing technologies and collaborative efforts between academia and industry, EVs hold great potential for advancing clinical treatments for alopecia in the future.

## Introduction

Alopecia is a common clinical disease that has a significant impact on patients’ quality of life and mental health [1]. According to whether the follicular opening is absent or not, alopecia can be divided into two categories: non-cicatricial alopecia and cicatricial alopecia. Androgenic alopecia, alopecia areata, telogen effluvium, and trichotillomania are common non-cicatricial alopecia types. Cicatricial alopecia can be divided into primary cicatricial alopecia (lichen planus, lupus erythematosus, folliculitis alopecia, etc.) and secondary cicatricial alopecia (secondary to trauma, burns, tumors, etc.). Genetic predisposition, changes in hormone levels, immune dysfunction, stress, infection, and trauma can all contribute to hair loss. Alopecia, especially cicatricial alopecia is difficult to detect and diagnose in its early stages, and the progression of the disease is gradually aggravated, which is usually accompanied by irreversible hair follicle damage, absence of hair follicles, skin fibrosis, and scarring.

**Figure 1.**
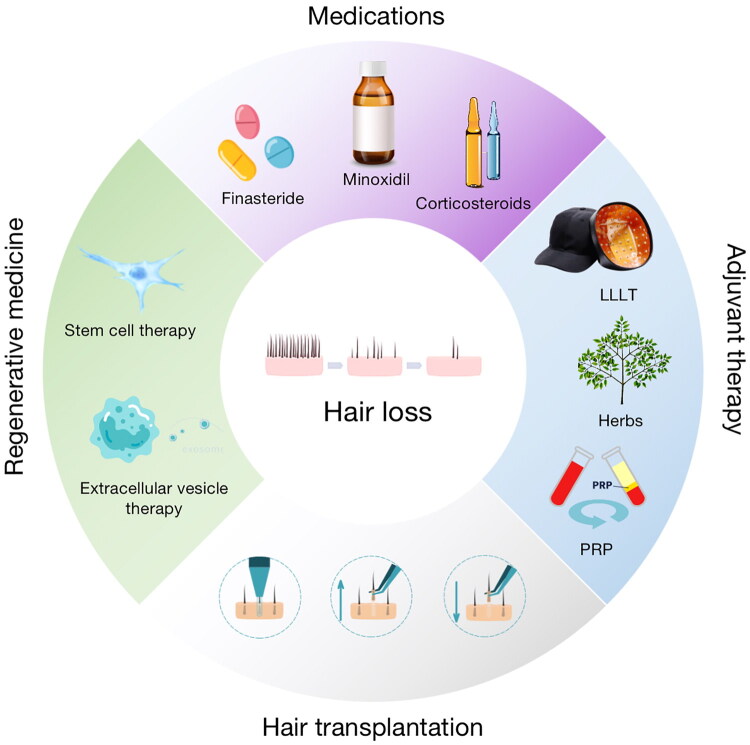
Schematic diagram of clinical treatment strategies for alopecia. PRP: platelet-rich plasma; LLLT: low level light therapy.

At present, the clinical treatment of alopecia mainly includes drug therapy, hair transplantation, low-level light therapy (LLLT), and stem cell therapy (Figure 1). Finasteride, minoxidil, glucocorticoids, and immunotherapy are the commonly used drugs for androgenic alopecia, female pattern hair loss, alopecia areata, and primary cicatricial alopecia [2–4]. However, the effect of drug treatment varies from person to person, the treatment course is long, hair loss will progress after discontinuation, and some patients may endure side effects, such as dermatitis, gynecomastia, and decreased libido [5–7]. Hair transplantation can effectively and continuously improve the appearance of hair loss effectively and continuously [8–10]. However, the donor areas for hair transplantation are limited [11,12]. Low-level light therapy (LLLT) with a wavelength of 635–678 nm, injection of platelet-rich plasma (PRP), or other mesotherapy have also been reported to improve hair loss [11–14]. Nevertheless, the parameters of treatment interval, optimal dose, and duration still require further verification, and their therapeutic effects still need to be validated in large-scale clinical trials [15,16]. Ideal treatment for alopecia is still lacked and research of new therapies is always desirable.

Regenerative medicine treatments based on tissue engineering and stem cell therapy have been applied in many fields, such as the reconstruction of damaged tissues and *in vitro* synthesis of skin. Some researchers have obtained patient scalp tissues by biopsy and injected cultured and expanded cells from scalp hair follicles into the hair loss area to stimulate hair growth. It is currently undergoing phase II clinical trials [17,18]. Adipose tissue, which is full of mesenchymal stem cells and a variety of growth factors, has been studied for the treatment of hair loss in various forms [19–21]. Human scalp epidermal stem cells and dermal papilla cells were co-cultured in acellular dermal scaffolds to prepare dermo-epidermal skin substitutes and then transplanted into full-layer skin wounds of nude mice to obtain structures similar to human hair follicles [22]. By cultivating fibroblasts, keratinocytes, and dermal papilla cells in a 3D printed mold that simulates the physiological structure of hair follicles and restores the complete transcription characteristics of dermal papilla cells (DPC) through overexpression of Lef-1, the structure of the human hair follicle can be obtained [23]. These studies indicate the possibility of using regenerative medical techniques to treat hair loss. Stem cells are abundant sources of self-renewal ability, multidirectional differentiation ability, and good therapeutic potential; however, the inherent heterogeneity and variability associated with cell expansion, risk of host immune rejection, potential tumorigenicity, and low integration rate with host cells limit the development of stem cell therapies.

Studies have shown that stem cells regulate tissue repair mainly through paracrine regulation, rather than through differentiation. Extracellular vesicles (EVs) are an important form of paracrine activity in stem cells. EVs can carry a variety of bioactive molecules, such as proteins, lipids, mRNA, miRNA, and DNA, and play a therapeutic role in the form of cell communication and transmission of endogenous substances and exogenous drugs [24–27]. Recently, many researchers have applied EVs to the study of hair growth and have made some progress [28–32]. Even though challenges, such as mass production and clinical application of EVs still remain, the approaches are still promising because the outcomes of previous studies are encouraging [33–39]. In this review, we will focus on the biological characteristics and preparation of EVs, the basic and clinical research of EVs in the treatment of hair loss, and the challenges of clinical transformation of EVs, thus providing some progress in the EVs treatment of alopecia.

## Methods

We conducted a comprehensive literature search in the PubMed database using a range of relevant keywords: extracellular vesicles, alopecia, hair loss, preparation, exosomes, microvesicles, apoptotic bodies, nanoscale particles, preclinical studies, clinical trial, clinical transformation, regenerative medicine, tissue engineering, stem cell, bioactive molecules, and extraction methods. The search was restricted to articles published up to July 2025. The papers mostly focus on the biological characteristics and preparation of EVs, the basic and clinical research of EVs in the treatment of hair loss, and the challenges of clinical transformation of EVs were retracted. Conference abstracts and papers written in a language other than English were excluded.

## Biological characteristics and preparation of extracellular vesicles

Recently, EVs have received considerable attention as an important component of intercellular communication [40]. EVs are spherical, nanoscale particles with a closed lipid bilayer structure. According to their formation mechanism and particle size, they can be divided into exosomes (30–150 nm in diameter), microvesicles (100–1000 nm in diameter), and apoptotic bodies (1000–6000 nm in diameter). EVs are widely detected in body fluids, such as blood, breast milk, saliva, semen, and urine [41]. They play a key role in intercellular communication by transporting miRNAs, proteins, lipids, and other biomolecules [42,43]. EVs-based therapy has recently been studied as a new alternative for cell therapy for the treatment of cancer, cardiovascular disease, wound healing, facial rejuvenation, and other diseases and symptoms [44–46]. Compared with cell therapy, EVs have the following advantages: (1) avoiding the immune risks caused by stem cell transplantation, (2) convenient storage conditions, (3) easy circulation through capillaries, and (4) reducing the risk of tumorigenicity [47]. In addition, the membrane structure of EVs can be genetically modified to express specific membrane proteins, and their lacuna-containing cell-like membrane topology can contain and deliver drug molecules; therefore, EVs are highly recognized as a promising biological tool in nanomedicine [48,49].

EVs can interact with target cells through three mechanisms: (1) direct integration of transmembrane proteins of EVs and signal receptors of target cells, (2) fusion of biomolecules carried by plasma membranes with the cytoplasm of recipient cells, and (3) internalization of extracellular vesicles into recipient cells [50]. Among the major components of EVs, miRNAs have received much attention because of their key roles in the regulation of gene expression. Through signal transduction, miRNAs can directly regulate the proliferation, migration, and differentiation of osteoblasts, mesenchymal stem cells, and chondrocytes and indirectly regulate the microenvironment and promote tissue repair [51]. In addition to miRNAs, long non-coding RNA (lncRNAs) can also be transferred to recipient cells by extracellular vesicles to participate in transcriptional and post-transcriptional regulation of biological processes, such as development, degeneration, and regeneration of cartilage [40,52,53]. The paracrine exchange of genetic information between mesenchymal stem cells (MSC) and target cells can also be realized by mRNA transport in EVs [54]. In addition, proteins and lipids in EVs can accelerate tissue regeneration through angiogenesis, immune regulation, and extracellular matrix remodeling [55,56].

EVs extraction methods include ultra-high-speed centrifugation, density gradient centrifugation, ultrafiltration, chemical precipitation based on vesicle solubility changes, and chromatography based on highly specific interactions between molecules and microfluidics on the vesicle surface [57]. Ultra-high-speed centrifugation is the most widely used separation method. Centrifugation was performed according to the molecular density and size, and the preparation time was 140–600 min. It has the advantages of a relatively simple preparation process and no need for the addition of exogenous chemical components. However, ultra-high-speed centrifugation usually takes a long time with high cost, and the purity and extraction scale are susceptible to the centrifugal time and rotational speed. They are mostly used in small-scale preparations [58,59]. Density gradient centrifugation was used to separate the EVs according to the preset density gradient. Compared to ultra-high-speed centrifugation, higher-purity vesicles can be obtained, and no extra chemical components are introduced. Density gradient centrifugation is also limited in preparative scale and time, thus limiting its clinical application [60]. Ultrafiltration filters vesicles through membranes with specific pore sizes, the operation of which is convenient with a high production efficiency and no extra components. The limitations of this technology are that the filter membrane is easy to clog, the sample preparation is of low purity, and the vesicles may leak, distort, or aggregate [61]. Size-exclusion chromatography can separate EVs according to the fluid mechanical volume, and the preparation process is mild, less polluted, and can prevent vesicles from aggregation. However, its preparation purity is also low, other molecules of the same size are doped, and the machine flow is small [62]. Polymer precipitation changes the solubility of vesicles by adding chemical reagents to the sample, which takes 8–12 h to prepare. This method can be used to prepare a high scale of vesicles at a low cost, but the prepared vesicles are usually mixed with chemical reagents that are added, and the preparation time is longer [63]. Anion exchange chromatography can be used to prepare EVs by charge separation in a short processing time and maintain the structure and biological integrity of the vesicles. However, this technique requires the additional separation of other charged biomolecules and sample concentration after preparation [64,65]. Repeatable, cost-effective, and high-throughput preparation methods are required to achieve clinical transformation, as the existing methods have the common drawback of a very low yield of EVs. Many researchers have tried to prepare biomimetic EVs with similar shapes, sizes, and potentials using engineering methods *in vitro* and *in vivo* to mimic natural EVs [66,67].

Technologies for the preparation of biomimetic EVs mainly include bottom-up and top-down strategies [49,68]. Bottom-up approaches typically bind special lipids with specific compositions that mimic exosomes into exosome-like nanoparticles and then synthesize lipid bilayers with the required surface proteins through simple incubation, chemobiological coupling, and cell-free protein synthesis [69]. Biomimetic EVs prepared in this way have controllable characterization, clear composition, and higher drug similarity than natural EVs. However, as the effective bioactive ingredients and delivery-related molecules in natural EVs are still unclear and the contents and surface markers vary with cell type and physiological state, it is difficult to fully replicate the nucleic acid, lipid, and protein composition of natural EVs [45,70]. The top-down approach involves splitting larger and more complex components to form smaller membrane fragment units and reassembling to form nanovesicles that mimic natural EVs [71]. The nanovesicles prepared in this way are almost the same as the source cells in terms of membrane structure and content composition, which can be prepared by microfluidic slices or mechanical extrusion [72,73]. Nevertheless, nanovesicles prepared in this manner are less controllable in terms of physical parameters and usually require additional purification steps [74].

## Basic and clinical study of extracellular vesicles in the treatment of alopecia

EVs play an important role in cell communication, tissue homeostasis, cell differentiation, organogenesis, and tissue remodeling [75]. The growth and cycle of hair are regulated by interactions between different cells, signaling pathways, and cytokines [76,77]. It has been suggested that EVs participate in the morphogenesis of hair follicles and changes in the hair cycle [78].

**Figure 2.**
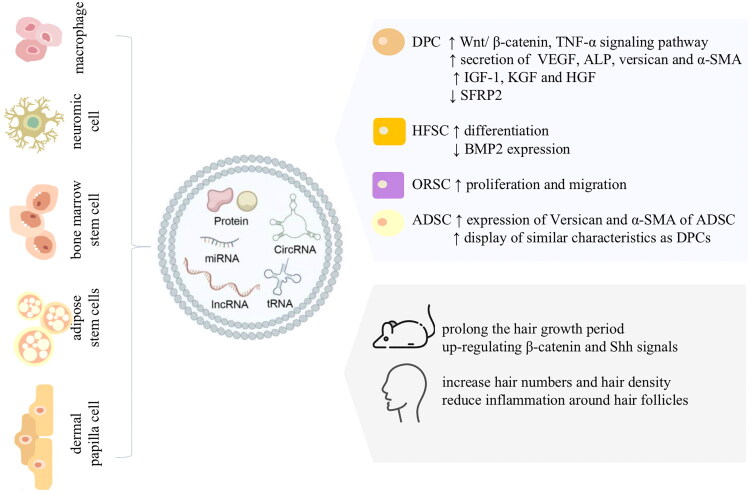
Diagram of mechanisms of extracellular vesicles in the treatment of alopecia. DPC: dermal papilla cells; HFSC: hair follicle stem cells; ORSC: outer root sheath cells; ADSC: adipose stem cells; VEGF: vascular endothelial growth factor; ALP: alkaline phosphatase; α-SMA: α-smooth muscle actin; IGF-1: insulin-like growth factor-1; KGF: keratinocyte growth factor; HGF: hepatocyte growth factor; SFRP2: secreted frizzled-related protein 2; BMP2: bone morphogenetic protein-2; Shh: sonic hedgehog.

Many preclinical studies have validated the role of EVs derived from different tissue sources (Figure 2). Exosomes of dermal papillary cells can induce the differentiation of hair follicle stem cells, promote the proliferation and migration of outer root sheath cells, promote the secretion of IGF-1, KGF, and HGF by DPC, prolong the hair growth period of mice by upregulating β-catenin and Shh signals, and delay hair entry into the degenerative stage [79]. The effect of DPC-derived exosomes on hair is influenced by the culture environment and cell passage. Compared with exosomes of DPC cultured in 2D culture, exosomes of cells cultured in a 3D environment could further downregulate the Wnt signal transduction inhibitor SFRP2 through overexpression of miR-218-5p, thus promoting hair regeneration. Compared with exosomes of passage 8 DPC, exosomes of passage 3 DPC showed stronger inhibition of BMP2 expression [80]. In another study, EVs derived from passage 1 DPC promoted hair follicle stem cell proliferation by targeting WIF1 through overexpression of miR-181a-5p [81]. Deng et al. observed that DPC exosomes can also improve hair loss in a mouse model of alopecia areata and reduce inflammation around hair follicles [82]. Treatment of adipose stem cells with DPC-EVs can promote the expression of versican and α-SMA in adipose stem cells (ADSC), leading to the display of characteristics similar to those of DPC [83]. Adipose stem cell-derived EVs have also been shown to promote DPC proliferation by upregulating the expression of Wnt/β-catenin, TNF-α signaling pathway, and VEGF, and to promote DPC expression of hair follicle-induced proteins, such as ALP, versican, and α-SMA [24]. In addition, macrophage-derived EVs, neuronic cell-derived EVs, bone marrow stem cell-derived exosomes, and dermal fibroblasts-derived EVs can also promote the proliferation of hair follicle cells and hair regeneration in mice [84–86].

At present, there are ten clinical trials using EVs to treat hair loss (Table 1); however, only four clinical trials have been completed. To date, only a few clinical studies have been published on this topic (Table 2). One published clinical trial was a prospective study including 30 male patients, aged between 22 and 65 years, with grade III-VI androgenetic alopecia according to the Norwood-Hamilton scale. The authors observed a statistically significant increase in hair density in the 4th and 12th weeks after treatment. The patients were more satisfied in the 12th week than in the 4th week. No severe side effects or complications were observed after the exosomes injection [33]. This is the first published clinical trial to show the inspiring effect of stem cells derived exosomes in the treatment of alopecia, but this study also had some limitations. Firstly, this is not a randomized controlled study with a placebo group. Secondly, the study does not evaluate the effects of repeated exosomes injections. There are other published prospective studies applying adipose stem cell-derived exosomes to treat alopecia [34,87]. Ester Lee et al. designed a 24-week, open-label, prospective study to compare hair regeneration effect before and after the treatment within the group [34]. In this study, a total of 30 participants aged from 18 to 60 years were recruited. The baseline mean hair density in the targeted area was 158.03/cm^2^. After 12 weeks, the mean hair density was 161.90/cm^2^ (*p* = 0.033), and after 24 weeks, it increased to 166.14/cm^2^. This study is the first translational research of adipose stem cell-derived exosomes in hair growth with long period of follow-up. However, this study is not a randomized controlled study with a placebo group. Wan J, et al. has performed a prospective, open-label study included 16 male patients aged 36–45 years with mild to moderate AGA (Norwood–Hamilton stages III–V). At the 12-month follow-up, there was a significant improvement in hair density among all patients [87]. This study has longer follow-up than the previous studies. But the sample size in this study was small and control group was lacked. The retrospective study has also showed that adipose-derived stem cells derived exosomes significantly improved the hair diameter in the hair loss area without adverse effects [36]. Human bone marrow mesenchymal stem cell-derived EVs were also found to be effective [35]. Although early studies suggested that EVs have some therapeutic effect on hair loss, future studies with longer follow-up periods, larger sample sizes, and randomized controlled trials are necessary to further validate the efficacy of EVs. At the same time, more clinical and preclinical studies are required to explore the mechanism.

**Table 1.** Clinical trials of extracellular vesicles in treatment of alopecia.

Clinical trials web	ID	Title	Study type	Enrollment number	Age range (years)	Status
ClinicalTrials.gov	NCT06571799	Study evaluating the efficacy and safety of BENEV exosome regenerative complex + for self-perceived thinning hair	Interventional	30	18–65	Completed
ClinicalTrials.gov	NCT05658094	Exosome effect on prevention of hairloss	Interventional	20	25–65	Unknown status
ClinicalTrials.gov	NCT06539273	Exosome treatment in androgenetic alopecia	Interventional	30	22–65	Completed [33]
ClinicalTrials.gov	NCT06482541	Efficacy and safety of AGE ZERO™ EXOSOMES to treat men and women with androgenetic alopecia	Interventional	100	More than 18	Not yet recruiting
ClinicalTrials.gov	NCT06239207	Efficacy and safety of exosomes *versus* platelet rich plasma in patients of androgenetic alopecia	Interventional	30	18–70	Completed
ClinicalTrials.gov	NCT066970/80	Umbilical cord-derived mesenchymal stem cell exosomes on hair growth in patients with androgenetic alopecia	Interventional	50	18–50	Active, not recruiting
International Clinical Trials registry platform	IRCT2 0200127046/282N54	Investigation of exosome injection in combination with minoxidil in patients with androgenic hair loss	Interventional	15	18–45	Recruiting
ClinicalTrials.gov	NCT069323/93	Exosomes for hairloss treatment	Interventional	18	18–75	Not yet recruiting
ClinicalTrials.gov	NCT069303/26	Exosome treatment and hair growth (exosome)	Interventional	24	20–50	Completed
ClinicalTrials.gov	NCT069994/08	Efficacy and safety of TargetCool + Benev exosomes in patients with hair thinning	Interventional	15	18–70	Not yet recruiting

**Table 2.** Studies utilizing extracellular vesicles for alopecia in humans.

Author	Study design	Condition	Sample size	Age (years)	Intervention	Duration	Outcomes	References
Mert Ersan, et al.	Prospective study	Androgenetic alopecia	30	22–65	Foreskin-derived mesenchymal stem cells derived exosomes	12 weeks	The mean hair density increased from 149.7 ± 13.7 hairs/cm^2^ at pre-treatment to 153.6 ± 16.8 hairs/cm^2^ at the 4th week (*p* = 0.043) and further to 157 ± 18.3 hairs/cm^2^ at the 12th week (*p* = 0.002).	[33]
Ester Lee, et al.	Prospective study	Androgenetic alopecia	30	18–60	Human adipose tissue stem cells derived exosomes	24 weeks	The baseline mean hair density in the targeted area was 158.03/cm^2^. After 12 weeks, the mean hair density was 161.90/cm^2^ (*p* = 0.033), and after 24 weeks, it increased to 166.14/cm^2^	[34]
Gordon H. Sasak	Retrospective study	Alopecia	31	27–87	Human bone marrow mesenchymal stem cell-derived extracellular vesicles	24 weeks	Details refer to the article	[35]
Byung-Soon Park, et al.	Retrospective study	Alopecia	39	20–66	Adipose-derived stem cells derived exosomes	12 weeks	Mean hair density increased from 121.7 ± 37.2 to 146.6 ± 39.5 hairs/cm^2^ (*p* < 0.001), and mean hair thickness increased from 52.6 ± 10.4 to 61.4 ± 10.7 μm (*p* < 0.001)	[36]
Suparuj Lueangarun, et al.	Case	Androgenetic alopecia	1	54	Rose stem cell-derived exosomes	3 months	The patient experienced significant hair regrowth, and photographic evaluations demonstrated considerable improvements after the sixth and twelfth sessions. Improvements in hair regrowth were also noted 3 months after the final treatment.	[37]
Wan J, et al.	Prospective study	Androgenetic alopecia	16	36–45	Adipose-derived stem cells derived exosomes	12 months	Patients with baseline densities of 75 hairs/cm^2^ had an increase to 110 hairs/cm^2^, while those with baseline densities of 95 hairs/cm^2^ had an increase to 125 hairs/cm^2^	[87]

In many studies, the main treatment form of EVs is subcutaneous injection, and because of their limited duration in the body, regular treatment is required to maintain the drug concentration and therapeutic efficacy. Yang et al. prepared water-soluble hyaluronic acid microneedle patches loaded with MSC-exosomes and the small-molecule drug UK5099, which was found to effectively promote hair growth at a low drug concentration [88]. Shiekh et al. designed an oxygen-releasing antioxidant wound dressing composed of polyurethane and loaded with ADSC-exosomes and promoted wound repair and hair follicle regeneration in rat wounds [89]. Chen et al. mixed extracellular vesicles (DPC-EVs) derived from dermal papilla cells with oxidized sodium alginate hydrogel and found that the hydrogel could delay the degradation of DPC-EVs and achieve continuous release, thus promoting the expression of MMP3 and Wnt3a mRNA and protein in hair cells and the growth of mouse hair [90]. At present, most of the EVs studies about promoting hair growth are preclinical studies. The potential mechanism, safety, and the most effective treatment plan (including the method of administration, cell source, and effective dose) of EVs therapy remain obscure, and more extensive and higher quality studies are required for further verification.

## Challenges in clinical transformation of extracellular vesicles

The role of EVs in regenerative medicine has been widely explored and has shown great therapeutic potential in various disease models [91]. Although EVs have been designed for clinical treatment in thousands of preclinical studies due to their unique properties, only a few EVs have been studied in clinical trials, and to date, there are no approved EVs-based therapies [92,93]. Efforts have been made to realize the industrial production of EVs that meet the requirements of clinical application. The production process of EVs includes upstream processing (cell isolation, storage and expansion, and culture medium preparation) and downstream processing (extraction, concentration, purification, and storage of EVs) [52]. Culture conditions, medium components, and donor cell passages all affect the biological characteristics; therefore, quality control of the production is particularly critical [94,95]. Most EVs preparation methods used in traditional laboratory studies are inconsistent with low yield, unstable composition, various therapeutic results, and low targeting efficiency, thus restricting the clinical transformation process [96–98]. Increasing the production of EVs, enhancing the biological activity by pretreatment, enhancing the function by drug loading, and targeting EVs by surface modification are still some of the challenges.

At present, methods to promote the production of EVs mainly include physical and non-physical methods. Physical methods include mechanical, geometric, acoustic, and electrical stimulation. Non-physical methods include molecular interference, environmental induction, and external inducer treatments [49,93]. Mechanical sensing is a fundamental pathway for cell-to-cell and cell-to-microenvironment communications [99]. The response of cells to mechanical loads can affect EVs signal secretion. Mechanical shear stress under direct current or turbulence induction could affect cell behavior and EVs secretion rate, and a low shear rate could stimulate 3D cultured human dermal vascular endothelial cells (HDMEC) to secrete extracellular vesicles [100]. Compared with the static control, the amount of EVs secreted by human skeletal muscle cells (HSKMC) in the cyclic stretching state was also significantly increased [101]. High-frequency ultrasound, 3D culture, hypoxic stress, and low-level electrical treatment can also significantly increase extracellular vesicle secretion. Compared with long-term and moderate exposure, transient but severe hypoxic exposure has a greater impact on exosome secretion [102]. The combination of sodium iodoacetate (IAA) and 2, 4-dinitrophenol (DNP) could inhibit glycolysis and oxidative phosphorylation, which would cause cell energy expenditure (decreased ATP levels and increased AMP levels) and stimulate adenosine release, thereby activating the A2B receptor system and ultimately leading to increased exosome production [103].

The composition of EVs is highly influenced by the genetic or phenotypic expression of the cells from which they are secreted. The properties of natural EVs vary according to the physiology of the cell and can be regulated by external stimuli [49]. Common preconditioning methods include (1) biochemical stimulation (e.g. pro-inflammatory cytokines, lipopolysaccharides, and nitric oxide), (2) modification of the cell culture environment (e.g. hypoxia, serum deprivation, and 3D culture), and (3) introduction of exogenous genes (e.g. microRNAs (miRNAs) and plasmid DNA). Different preconditioning methods of thrombin, hypoxia, lipopolysaccharides, and hydrogen peroxide could enhance the efficacy of MSC-EVs in promoting wound healing through the extracellular signal-regulated protein kinase (pERK1/2) and pAKT signaling pathways [104,105]. EVs of MSCs pretreated with 455 nm blue light could up-regulate the expression of miR-135-5p and miR-499-3p and promote angiogenesis in animal models of deep second-degree burn [106]. EVs isolated from MSCs treated with TNF-α contain a variety of neuroprotective factors, such as PEDF, VEGF-A, and PDGF-AA, which can promote the proliferation of retinal ganglion cells from rodents and humans [107]. In the LPS-mediated inflammatory microenvironment, dental cell-derived EVs can promote the proliferation, migration, and differentiation of periodontal membrane cells, upregulate the expression of proteins related to osteogenesis and adhesion, and induce periodontal regeneration in periodontitis in rats [108].

Secreted EVs can facilitate intercellular communication through internalization, during which biomolecules, such as proteins or mRNA are transferred and induce responses in the target cell. Owing to their ability to cross tissue and cellular barriers, EVs have been studied as biocompatible delivery vehicles for chemicals, proteins, and genetic drugs. EVs are also targeted to specific cell or tissue types [49]. There are two ways to load therapeutic drugs into EVs: (1) endogenous loading of drugs into EVs-derived cells, which then secrete drug-loaded EVs, and (2) exogenous loading of drugs into isolated EVs. Chen et al. used miR-146a simulated vector to transfect adipose-derived mesenchymal stem cells and extracted exosomes, which enhanced the proliferation and migration of NIH/3T3 cells by activating the serine protease inhibitor family H member 1 (SERPINH1) and pERK signaling pathways [109]. Didiot et al. loaded hydrophobically modified small interfering RNA (hsiRNA) into exosomes to silence Huntington protein mRNA and protein, and the results showed that when exosomes containing hsiRNA were unilaterally injected into the mouse striatum, Huntington gene silencing was more significant than when hsiRNA was injected alone [110]. Endogenous methods can easily load biomolecules into exosomes, but their secretion and loading are limited by the physiology of the source cell; thus, they cannot be customized, quantified, or modified [111]. In exogenous loading, EVs can be loaded with specific types and quantities of biomolecules that are not limited by the variety and physiological characteristics of the cell [112]. At present, loading and delivery through EVs still have a low loading efficiency and are limited to hydrophobic drugs. Electroporation, saponin incubation, ultrasonic treatment, extrusion, low permeability analysis, freeze-thaw cycles, and pH neutralization can enhance the membrane permeability of EVs and improve the loading efficiency of drugs [113,114].

Improving the targeting ability of EVs using endogenous or exogenous methods could improve the therapeutic specificity of EVs to specific tissues. Endogenous methods include transfection of the expression vector containing the gene encoding the targeted peptide that is part of the EVs surface into the EVs-derived cells through genetic engineering, so that the EVs surface expresses the targeted peptide or ligand with low transfection efficiency [66]. Bioorthogonal chemistry has also been applied to the endogenous surface modification of EVs, treating the cell with a lipid or mannosamine derivative that could display an azide group on the EVs surface and then achieving surface modification by introducing an acetylene containing a functional part. Bioorthogonal chemistry has the advantages of higher efficiency and repeatability, but high concentrations of bioorthogonal chemical reactants have certain cytotoxicity, which might lead to changes in the EVs composition [115]. External surface modifications can be performed directly on EVs using physical or chemical methods. Physical methods include ultrasonic treatment, extrusion, and freeze-thaw, which can alter the surface properties of EVs through membrane rearrangement. Chemical methods used include phospholipid insertion, bioconjugation, and click chemistry [116–118]. Vandergriff et al. prepared infarct-targeting exosomes by binding the cardiac homing peptide (CHP) to the surface of CDC exosomes using DOPE-NHS splices. After intravenous administration, CHP-bound exosomes (CHP-XO) were detected, and the retention time of CHP-XO in the heart was prolonged. It can also effectively improve myocardial ischemia in ischemia/reperfusion injury models [119]. Tian et al. inserted the c(RGDyK) peptide onto the surface of exosomes (cRGD-Exo) through bioorthogonal chemistry to target the delivery of curcumin to treat ischemic injury. After systemic administration in a mouse model of transient middle cerebral artery occlusion, cRGD-Exo loaded with curcumin effectively inhibited inflammation and apoptosis [120].

Recent studies have demonstrated that functionalized EVs have great therapeutic potential. However, there are still many considerations and technical processes that need to be solved from the experimental to the clinical stage, such as how pretreated cells can generate cellular stress that affects the composition of EVs, how to separate drug-loaded and non-drug-loaded EVs for purification, and surface modification strategies that may interfere with the inherent function of EVs. Bionic nanovesicles can solve some of the challenges associated with the production and purification of natural EVs. However, bionic nanovesicles also have problems with homogeneous production and clear characterization of the components. In addition, the clinical application of EVs as drugs requires further development of cost-effective large-scale production technology, measurement methods of product concentration, quality control standards, and long-term safety concerning EVs products.

## Conclusion

EVs-based therapy has been widely studied as a promising therapeutic method for the treatment of cancer, cardiovascular disease, wound healing, facial rejuvenation, and other diseases. Recently, many scholars have focused on the effect of EVs on the treatment of hair loss and have made some progress. However, this method is still far from being clinically used. Further research should be conducted to optimize dosing, cell sources of EVs, and frequency of administration for more effective and safer therapies. At present, most studies of EVs in humans are retrospective and simple prospective studies, while results of randomized controlled studies have not been conducted. Further randomized controlled clinical trials should be conducted to validate the effectiveness of EVs in the treatment of alopecia. It is believed that in the future, through innovative advances in manufacturing technology and active academic and industry collaborations, these limitations will be overcome and substantial progress will be made in the application of EVs for clinical therapy.

## Data Availability

Data sharing is not applicable to this article as no data were created or analyzed in this study.
